# Wearable saving lives: a case report

**DOI:** 10.1016/j.resplu.2026.101447

**Published:** 2026-08-12

**Authors:** Catharina E. Jansen, Jeroen Jaspers Focks, Sunder Manglani Rodriguez, Lente Pol, Roos Edgar, Niels van Royen, Judith L. Bonnes

**Affiliations:** a Department of Cardiology, Radboud University Medical Center, Geert Grooteplein Zuid 10, 6525 GA Nijmegen, the Netherlands; b Department of Cardiology, Slingeland Hospital, Kruisbergseweg 25, 7009 BL Doetinchem, the Netherlands

**Keywords:** Out-of-hospital cardiac arrest, Wearable, Fall detection

## Abstract

**Introduction:**

Unwitnessed cardiac arrests account for approximately half of all out-of-hospital cardiac arrests (OHCAs) and are associated with poor survival due to delayed recognition. Sudden physical collapse is a key characteristic of cardiac arrest and may be detected using accelerometer-based fall detection algorithms incorporated into commercial wearable devices. We report a case of unwitnessed OHCA in which smartwatch-based fall detection triggered automated alerts, resulting in timely initiation of cardiopulmonary resuscitation and favourable outcome.

**Case description:**

A 69-year-old man suffered an unwitnessed OHCA while running alone. Wearing an Apple Watch with fall detection activated, his collapse triggered automated alerts to emergency contacts and emergency medical services (EMS). Family members rapidly reached the patient, found him unresponsive, and initiated CPR approximately 6–7 min after collapse. EMS arrived shortly thereafter and documented ventricular fibrillation as the initial rhythm. Following advanced life support, return of spontaneous circulation was achieved. Hospital investigations revealed severe two-vessel coronary artery disease with an aberrant right coronary artery with interarterial course and prior infarctions in the RCA and LAD territories. The patient underwent coronary artery bypass grafting and received an implantable cardioverter-defibrillator for secondary prevention. The patient was discharged home 29 days later with complete neurological recovery.

**Discussion:**

This case illustrates the potential of wearable devices to serve as digital witness in otherwise unwitnessed cardiac arrests. It further highlights the promise of dedicated wearable cardiac arrest detection technologies that may reduce delays in recognition and treatment, thereby potentially improving survival and neurological outcomes.

## Introduction

Survival after out-of-hospital cardiac arrest (OHCA) depends on rapid recognition and timely initiation of cardiopulmonary resuscitation (CPR).^1^, ^2^ This is particularly challenging in unwitnessed cardiac arrests, which account for approximately half of all OHCAs and are associated with poor survival due to delayed recognition and activation of the chain of survival.^3^ To address this challenge, wearable technologies capable of automatically detecting cardiac arrest and alerting emergency responders are under active development. However, many commercially available wearables already incorporate accelerometry-based fall detection algorithms. As sudden collapse is a common manifestation of cardiac arrest, these devices may also facilitate earlier recognition of unwitnessed OHCA.^4^ We present a case of an unwitnessed OHCA during running, in which timely CPR was initiated after an Apple Watch detected the collapse and alerted family members.

## Case description

A 69-year-old man with a medical history of bronchiectasis and asthma was running on a dead-end road at the edge of town. He was out of sight behind a hedge and was wearing his Apple Watch (Apple Watch 10) on his left wrist. Fall detection functionality was activated,^5^ as it routinely was. Without preceding symptoms, he suffered an unwitnessed OHCA and collapsed to the ground. The Apple Watch detected the fall and automatically alerted predefined emergency contacts as well as the emergency medical services by calling 112.

**Fig. 1:**
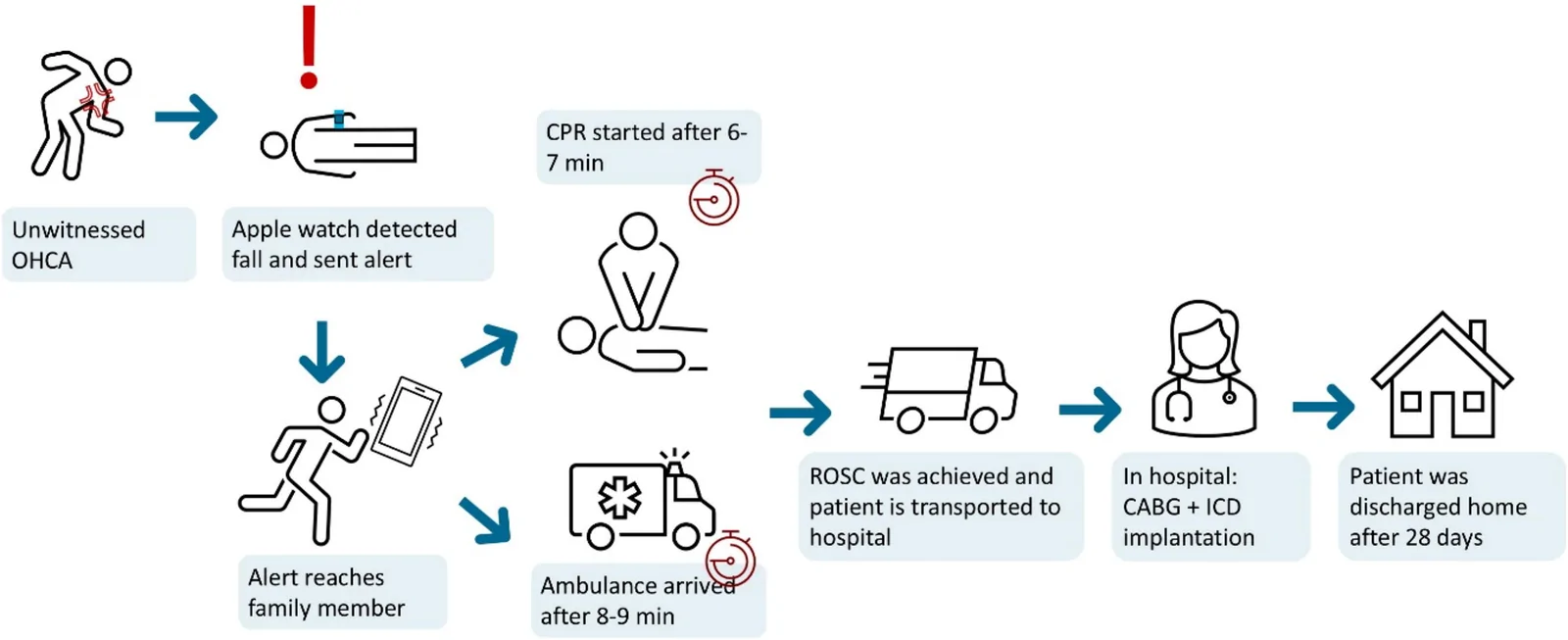
**Chain of survival initiated by the wearable triggered fall alert**.

Family members (his emergency contacts) went to the patient, found him unresponsive, and immediately initiated CPR (i.e. approximately 6–7 min after collapse; Fig. 1). Given the remote/hidden location no bystanders were present. Emergency medical services arrived about 1.5 min later. Initial rhythm analysis revealed ventricular fibrillation (VF), for which four defibrillation shocks were delivered. In addition, 3 mg adrenaline and 300 mg amiodarone were administered, after which return of spontaneous circulation was achieved.

On hospital arrival, he was hemodynamically stable, with a blood pressure of 140/60 mmHg and a heart rate of 60 beats/min; no external injuries were observed. The Glasgow Coma Scale (GCS) score was 3 with bilaterally reactive pupils. Electrocardiography demonstrated sinus rhythm with left axis deviation, an atypical right bundle branch block pattern, and diffuse ST-segment depression without ST-segment elevation. Bedside echocardiography showed reduced left ventricular function with hypokinesia of the inferoseptal and inferior wall; there was no right ventricular dilatation or pericardial effusion. Cranial CT showed no intracranial haemorrhage. Arterial blood gas analysis revealed severe metabolic acidosis (pH 7.15, lactate 8 mmol/L). Initial high sensitivity cardiac troponin T level was 36 ng/l.

With a presumed diagnosis of non-ST-elevation myocardial infarction (NSTEMI), the patient was admitted to the intensive care unit, where he received mechanical ventilation, inotropic and vasopressor support, as well as dual antiplatelet therapy. Maximum hs-cTnT concentration was 399 ng/l. The patient was successfully extubated on day 3 after admission. Initially, signs of post-anoxic encephalopathy were present; however, neurological status gradually improved, and he was transferred to the cardiology ward on day 6 with a GCS score of 15 (E4M6V5).

**Fig. 2:**
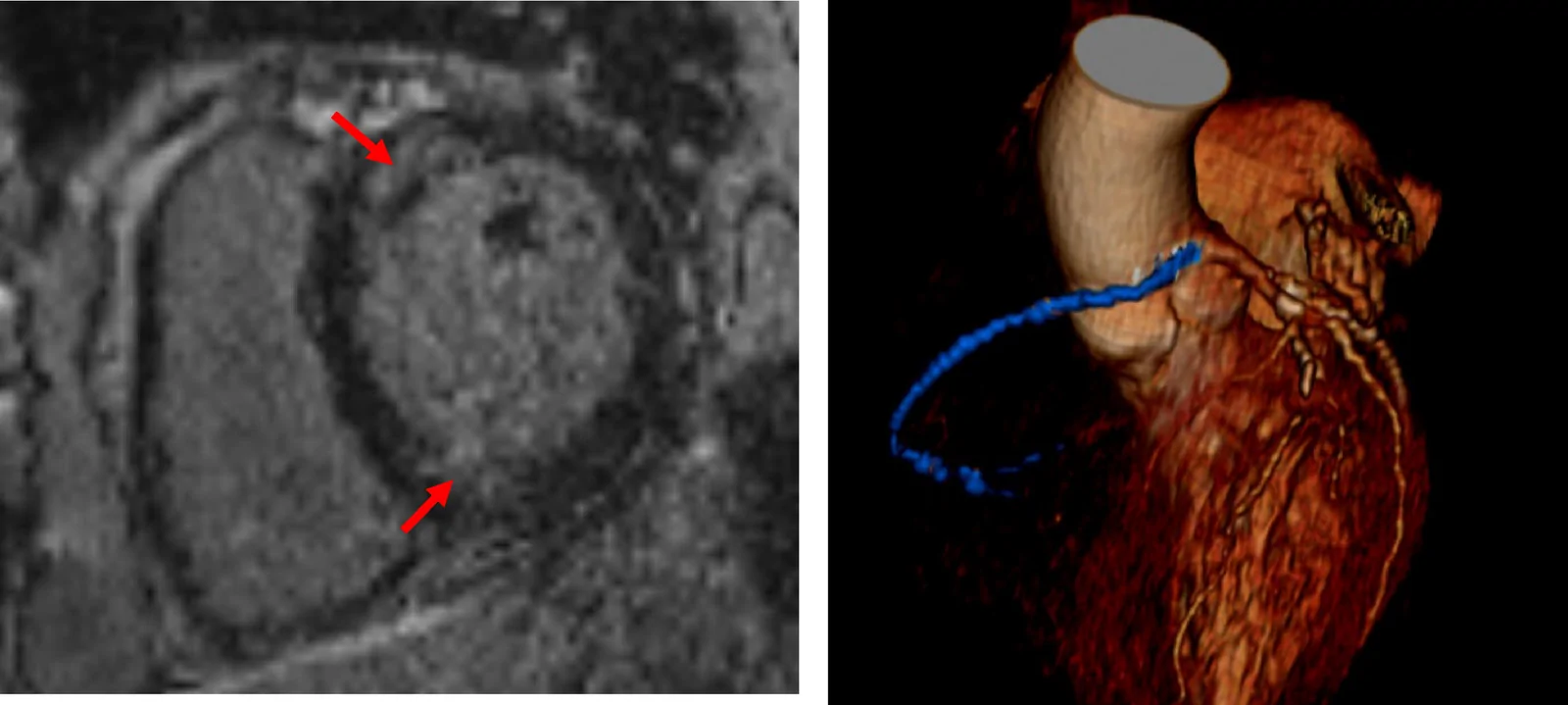
**CMR and 3D reconstruction of CT images showing previous infarctions in RCA and LAD territories and an aberant course of the right coronary artery**. Left: CMR late gadolinium enhancement image showing a short axis view of the left and right ventricle at the basal level with subendocardial LGE basal inferoseptal, anteroseptal, and anterior wall segments (red arrows) consistent with prior infarctions in the RCA and LAD territories. Right: 3D reconstruction of cCTA images of the right coronary artery (RCA, in blue) demonstrating a slit-like origin with a malignant interarterial course between the aortic root and the proximal pulmonary trunk (images made in TeraRecon Intuition Foster City, USA). 3D; three dimensional, CMR: cardiac magnetic resonance, cCTA: coronary computed tomography angiography LGE: late gadolinium enhancement. (For interpretation of the references to colour in this figure legend, the reader is referred to the web version of this article.)

On day 7, a coronary angiography (CAG) was performed and revealed a chronic total occlusion of an aberrant right coronary artery (RCA) and an approximately 80% stenosis of the proximal left anterior descending artery (LAD), with a subtotal stenosis at the level of the second diagonal branch. To further visualise the course of the RCA, a coronary computed tomography angiography (cCTA) was performed. The cCTA revealed that the RCA originated anteriorly from the left coronary cusp and followed an interarterial course between the aortic root and the pulmonary trunk (Fig. 2). Given the presence of severe two-vessel coronary artery disease and a malignant course of the RCA, coronary artery bypass grafting (CABG) was performed. On day 22, the patient underwent CABG with a left internal mammary artery (LIMA) graft to the LAD and saphenous grafts to the diagonal branch, the anterolateral branch and to the RCA (LIMA-to-LAD, vein-to-D-AL, vein-to-RCA). Except for short-lasting atrial fibrillation, the subsequent hospital course was uncomplicated.

Cardiac magnetic resonance imaging demonstrated mildly reduced left ventricular systolic function with subendocardial late gadolinium enhancement in the basal inferoseptal, anteroseptal, and anterior wall segments, consistent with prior myocardial infarctions in the RCA and LAD territories (Fig. 2). Given the final diagnosis of VT/VF related to a previous myocardial infarction, the patient met the criteria for a secondary prevention implantable cardioverter-defibrillator (ICD), which was implanted on day 28.

The patient was discharged home on day 29 after the OHCA and underwent cardiac rehabilitation. The patient made a full neurological recovery without residual deficits.

Written informed consent was obtained from the patient for publication of this case report and any accompanying images or clinical data.

## Discussion

This case illustrates the potential of wearable devices to act as digital witnesses in otherwise unwitnessed OHCA. Following sudden collapse, smartwatch-based fall detection automatically alerted both emergency contacts and emergency medical services, resulting in early recognition of the event, timely initiation of CPR, and a favourable neurological outcome.

Given the poor prognosis of unwitnessed OHCA, with reported survival rates below 5%, reducing delays in recognition and treatment remains of utmost importance.^6^ Several groups are currently developing dedicated wearable technologies capable of automatically detecting cardiac arrest and activating the emergency medical response system.^7^, ^8^, ^9^ These technologies primarily rely on photoplethysmography (PPG) to detect the absence of pulsatile blood flow at the wrist, while accelerometry has been incorporated as a complementary sensor modality to account for ongoing movement and reduce false positive cardiac arrest detections. However, the present case demonstrates that accelerometery may also play a direct role in detecting cardiac arrest. In this patient, collapse detection by an accelerometer-based fall detection algorithm triggered the emergency response pathway despite the absence of a dedicated cardiac arrest detection algorithm. Furthermore, a previous study in healthy volunteers demonstrated that a machine-learning algorithm based on accelerometery could detect simulated cardiac arrest-related collapses with high sensitivity and low false positives.^10^ Together, these findings suggest that motion and posture data derived from accelerometery may provide valuable complementary information to PPG, potentially improving the performance and reliability of future wearable cardiac arrest detection systems.

While wearable devices and algorithms specifically designed to detect cardiac arrest are still largely under development, this case demonstrates that currently available consumer wearables may already provide clinical value by facilitating earlier recognition of unwitnessed cardiac arrests. This applies not only to fall detection systems, as illustrated in the present case, but also to loss-of-pulse detection functionality available in selected smartwatch models. Although the sensitivity and real-world effectiveness of these technologies require further evaluation, this case highlights the importance of awareness and appropriate activation of available safety features, particularly in individuals at increased risk of cardiac arrest.

Importantly, wearable-based cardiac arrest detection alone is unlikely to improve outcomes unless it is coupled with a rapid emergency response. The potential survival benefit of wearable cardiac arrest detection technologies therefore depends not only on detection accuracy, but also on the ability of emergency contacts and EMS to act promptly on generated alerts. To ensure that EMS can respond effectively to all alerts, false-positive rates must remain low. This case demonstrates that such a response pathway is feasible and can result in favourable clinical outcomes. A favourable factor in this case was that the cardiac arrest occurred in a public location, allowing emergency contacts and EMS to reach the patient without delay. In other situations, such as when a cardiac arrest occurs in a locked residence, additional barriers to patient access may reduce the benefit of automated detection and alerting.

## Conclusion

This case demonstrates that smartwatch-based fall detection can facilitate early recognition of otherwise unwitnessed cardiac arrest by automatically alerting emergency contacts and emergency medical services following sudden collapse. The resulting rapid response enabled timely CPR and successful resuscitation with complete neurological recovery. While dedicated cardiac arrest detection technologies are still under development, currently available wearable devices may already contribute to the chain of survival.

## CRediT authorship contribution statement

**Catharina E. Jansen:** Conceptualization, Data curation, Investigation, Methodology, Project administration, Validation, Visualization, Writing – original draft, Writing – review & editing. **Jeroen Jaspers Focks:** Conceptualization, Data curation, Supervision, Validation, Writing – review & editing. **Sunder Manglani Rodriguez:** Conceptualization, Supervision, Writing – review & editing. **Lente Pol:** Conceptualization, Investigation, Validation, Writing – review & editing. **Roos Edgar:** Conceptualization, Validation, Writing – review & editing. **Niels van Royen:** Conceptualization, Supervision, Writing – review & editing. **Judith L. Bonnes:** Conceptualization, Data curation, Investigation, Methodology, Project administration, Supervision, Validation, Visualization, Writing – original draft, Writing – review & editing.

## Ethics approval and consent to participate

The patient provided written informed consent for publication of this case report.

## Funding

None related to this case report.

## Data availability

No new datasets were generated or analysed for this case report.

## Declaration of competing interest

The author is an Editorial Board Member/Editor-in-Chief/Associate Editor/Guest Editor for Resuscitation Plus and was not involved in the editorial review or the decision to publish this article.

The authors declare the following financial interests/personal relationships which may be considered as potential competing interests: Roos Edgar received a research grant from the European Resuscitation Council not related to this manuscript; is editorial board member of Resuscitation Plus. Niels van Royen received a research grant from the Dutch Heart Foundation and Radboud University Medical Centre not related to this manuscript; received research grants from Biotronik, Abbott, Medtronic, and Philips, not related to this manuscript; received speaker fees from Abbott and Bayer. Judith L. Bonnes received a research grant from ZonMw not related to this manuscript. All other authors declare no competing interests.

## References

[1] McBride O., Poel A., Counts C.R. (2025). Temporal patterns in out-of-hospital cardiac arrest incidence and outcome. JAMA Cardiol.

[2] Waalewijn R.A., de Vos R., Tijssen J.G., Koster R.W. (2001). Survival models for out-of-hospital cardiopulmonary resuscitation from the perspectives of the bystander, the first responder, and the paramedic. Resuscitation.

[3] Hostler D., Thomas E.G., Emerson S.S. (2010). Increased survival after EMS witnessed cardiac arrest. Observations from the Resuscitation Outcomes Consortium (ROC) epistry-cardiac arrest. Resuscitation.

[4] Hutton J., Puyat J.H., Asamoah-Boaheng M. (2023). The effect of recognition on survival after out-of-hospital cardiac arrest and implications for biosensor technologies. Resuscitation.

[5] Apple. Use fall detection with apple watch; 2026. https://support.apple.com/en-us/108896 [accessed 2026-7-22].

[6] Nguyen D.D., Spertus J.A., Kennedy K.F. (2024). Association between delays in time to bystander CPR and survival for witnessed cardiac arrest in the United States. Circ Cardiovasc Qual Outcomes.

[7] Hutton J., Lingawi S., Puyat J.H. (2022). Sensor technologies to detect out-of-hospital cardiac arrest: a systematic review of diagnostic test performance. Resusc Plus.

[8] Shah K., Wang A., Chen Y. (2025). Automated loss of pulse detection on a consumer smartwatch. Nature.

[9] van den Beuken W.M.F., Niderost B., Goossen S.A. (2025). Automated cardiac arrest detection and emergency service alerting using device-independent smartwatch technology: proof-of-principle. Resuscitation.

[10] Edgar R., Ebrahimkheil K., Meeuwsen D. (2026). A machine learning model to detect falls mimicking cardiac arrest-related collapse based on wrist-derived accelerometry: the DETECT-2 study. Eur Heart J Digit Health.

